# Cardiac-respiratory self-gated cine ultra-short echo time (UTE) cardiovascular magnetic resonance for assessment of functional cardiac parameters at high magnetic fields

**DOI:** 10.1186/1532-429X-15-59

**Published:** 2013-07-04

**Authors:** Verena Hoerr, Nina Nagelmann, Arno Nauerth, Michael T Kuhlmann, Jörg Stypmann, Cornelius Faber

**Affiliations:** 1 Department of Clinical Radiology, University Hospital Muenster, Muenster, Germany; 2 Bruker BioSpin MRI GmbH, Ettlingen, Germany; 3 European Institute for Molecular Imaging, Muenster, Germany; 4 Department of Cardiovascular Medicine, Division of Cardiology, University Hospital Muenster, Muenster, Germany

**Keywords:** High magnetic field, Flow artifacts, Cardiovascular magnetic resonance, Retrospective gating, Self-gated cine UTE

## Abstract

**Background:**

To overcome flow and electrocardiogram-trigger artifacts in cardiovascular magnetic resonance (CMR), we have implemented a cardiac and respiratory self-gated cine ultra-short echo time (UTE) sequence. We have assessed its performance in healthy mice by comparing the results with those obtained with a self-gated cine fast low angle shot (FLASH) sequence and with echocardiography.

**Methods:**

2D self-gated cine UTE (TE/TR = 314 μs/6.2 ms, resolution: 129 × 129 μm, scan time per slice: 5 min 5 sec) and self-gated cine FLASH (TE/TR = 3 ms/6.2 ms, resolution: 129 × 129 μm, scan time per slice: 4 min 49 sec) images were acquired at 9.4 T. Volume of the left and right ventricular (LV, RV) myocardium as well as the end-diastolic and -systolic volume was segmented manually in MR images and myocardial mass, stroke volume (SV), ejection fraction (EF) and cardiac output (CO) were determined. Statistical differences were analyzed by using Student *t* test and Bland-Altman analyses.

**Results:**

Self-gated cine UTE provided high quality images with high contrast-to-noise ratio (CNR) also for the RV myocardium (CNR_blood-myocardium_ = 25.5 ± 7.8). Compared to cine FLASH, susceptibility, motion, and flow artifacts were considerably reduced due to the short TE of 314 μs. The aortic valve was clearly discernible over the entire cardiac cycle. Myocardial mass, SV, EF and CO determined by self-gated UTE were identical to the values measured with self-gated FLASH and showed good agreement to the results obtained by echocardiography.

**Conclusions:**

Self-gated UTE allows for robust measurement of cardiac parameters of diagnostic interest. Image quality is superior to self-gated FLASH, rendering the method a powerful alternative for the assessment of cardiac function at high magnetic fields.

## Background

Cardiovascular magnetic resonance (CMR) is increasingly often performed at very high magnetic field strength, up to 7 T for human subjects and up to 17.6 T for small animal models [1-7]. While higher magnetic fields hold the promise of providing better signal-to-noise ratio (SNR), higher spatial resolution or better dynamics, several technical issues remain. Apart from the typical high field challenges such as magnetic field inhomogeneities and specific absorption rate limitations, two major problems must be addressed for CMR in small animal models in particular: artifacts in the electrocardiogram (ECG) generated by the MR scanner itself and dynamic susceptibility artifacts derived from blood flow. To overcome problems with disturbed ECG signal retrospective gating strategies have been developed [8-10]. A very efficient approach is to use self-gating acquisition schemes, which do not require additional scans to record reference data and thus do not increase scan time. These techniques, however, do not mitigate the second crucial issue, flow artifacts. Reference data which have to be recorded during echo formation in self-gating strategies may even prolong the minimum echo time (TE) and thus lead to pronounced flow artifacts and susceptibility effects at the myocardium-lung interface. Flow artifacts may compromise exact measurement of the ventricular volumes or proper visualization of the valves. Susceptibility artifacts often impede quantification of right ventricular (RV) mass, which is already difficult due to lower contrast to other tissues at high magnetic fields.

One option to overcome these problems is to perform CMR with methods which allow for minimized TE [11]. Data acquisition with direct read out can be achieved with the technique termed ultra-short echo time (UTE) CMR. UTE sequences use center-out radial data acquisition schemes, directly recording signal after the excitation pulse [12]. Besides MR of tissue with short T_2_[13,14], UTE has previously been used for example for flow mapping of high velocity flow [11], MR angiography [15] or detection of iron oxide nano particles [16,17]. Although no echo is formed in UTE we will use the term TE for the delay between excitation and start of acquisition, according to established conventions.

A self-gated version of radial data acquisition has previously been proposed and shown to be able to compete in terms of SNR and contrast-to-noise ratio (CNR) with both ECG-triggered acquisition [18] and cartesian acquisition schemes [19]. Here, we have implemented and optimized a version of self-gated UTE on a 9.4 T small animal CMR scanner. We quantitatively and qualitatively compared the self-gated UTE sequence with a self-gated fast low angle shot (FLASH) sequence and with echocardiography in a mouse model of normal cardiac function.

## Methods

### Animals

Experiments were performed on six 12 weeks old (22 ± 1 g) healthy female C57BL/6 wild-type mice, purchased from Harlan-Winkelmann (Borchen, Germany). All animal studies were performed with the approval of the State Review Board of North Rhine-Westphalia, Germany (Az. 87–51.04.2011.A003). Mice were imaged using self-gated cine FLASH (IntraGate, Bruker BioSpin MRI, Ettlingen, Germany) and self-gated cine UTE sequences to assess the two imaging sequences with respect to CNR and cardiac functional parameters. As comparison, mice were also measured by ultrasound.

### In vivo ultrasound measurements

Cardiac Doppler-Echocardiography was performed on a specialized ultrasound biomicroscope for examination of murine models with high temporal (up to 1000 pictures per second) and spatial resolution (down to 35 μm) (VEVO 2100, VisualSonics, Toronto, Canada). Examinations were performed as previously described [20,21]. In brief, after anesthesia with isoflurane (2.0% isoflurane/98% O_2_) mice were shaved precordially using a commercially available electro-shaver and secured in a supine position on the heating pad of the VEVO 2100 with a feedback rectal body heat control holding a temperature of 38°C and controlling one-lead electrocardiogram heart rate above 400 bpm constantly for the normal scanning time of 20 minutes. Warmed and centrifuged gel was applied to the chest of the examined mice and examinations were performed with the system’s 25 MHz linear probe. ECG-triggered parasternal short axis views of the whole mouse heart were obtained in steps of 100 μm together with a parasternal long axis view. Routinely pulsed wave Doppler signals of the velocity time integral of the mitral valve inflow, the aortic outflow and the pulmonic outflow were obtained.

### Ultrasound Image analysis

Ultrasound image analysis was performed independently by two experienced ultrasound readers using the VEVO 2100 implemented image analysis. 2D images were manually segmented for end-diastolic and end-systolic heart cycle. Velocity time integrals of the valvular blood flow signals were manually segmented, too. Calculations of the volumes followed Teichholz’s formula (m-mode) and Simpson’s formula (2D). Calculations of cardiac output (CO), stroke volume (SV) were based on either the 2D measurements or the continuous flow formula.

### In vivo CMR measurements

CMR images of healthy C57BL/6 mice were acquired at 9.4 T on a Bruker BioSpec 94/20 (Ettlingen, Germany) equipped with a 1 T/m gradient system and a 35 mm volume coil. The MR system was interfaced to a console running ParaVision software 5.1 including the IntraGate software for sequence acquisition and reconstruction. For MR acquisition a cardiac and respiratory self-gated UTE sequence was implemented and animals were imaged with the following parameters: slice thickness, 1 mm; number of slices, 9; matrix size, 156 × 156; field of view, 20 × 20 mm^2^; averages, 1; TE, 0.314 ms; TR, 6.2 ms; flip angle, 15°; pulse shape, gaussian; pulse length, 0.3 ms; number of projections, 246; polar undersampling, 2.0; number of movie cycles, 200; acquisition bandwidth, 100 kHz; scan time per slice, 5 min 5 sec. No half-pulse was used. Gradients were ramped in a 160-μs delay, which allowed for gradient rising to full amplitude (115 μs, 128 μs, 132 μs for the three axes) and preemphasis corrections. K-space was sampled with constant increments of the backprojection angle. After the acquisition a delay of 2.5 ms was applied to achieve the same TR as in the FLASH sequence. For reconstruction, interpolation of the radially sampled k-space data onto a Cartesian grid was performed by a Kaiser Bessel Kernel regridding algorithm [22-24]. A stack of contiguous short-axis slices was acquired to cover the entire right and left ventricles. Image quality as well as functional and morphological cardiac parameters were assessed and compared to those obtained by using the self-gated cine FLASH (IntraGate FLASH) sequence with the following parameters: slice thickness, 1 mm; number of slices, 9; matrix size, 232 × 232; field of view, 30 × 30 mm^2^; averages, 1; TE, 3 ms; flip angle, 15°; pulse shape, gaussian; pulse length, 0.5 ms; TR, 6.2 ms; number of repetitions, 200; acquisition bandwidth, 75 kHz; scan time per slice, 4 min 49 sec.

During CMR measurements, animals were anesthetized with 2% isoflurane and were monitored for core body temperature and respiration rate using a CMR compatible monitoring system (SA Instruments, Stony Brook, NY).

### CMR techniques and reconstruction

**Figure 1:**
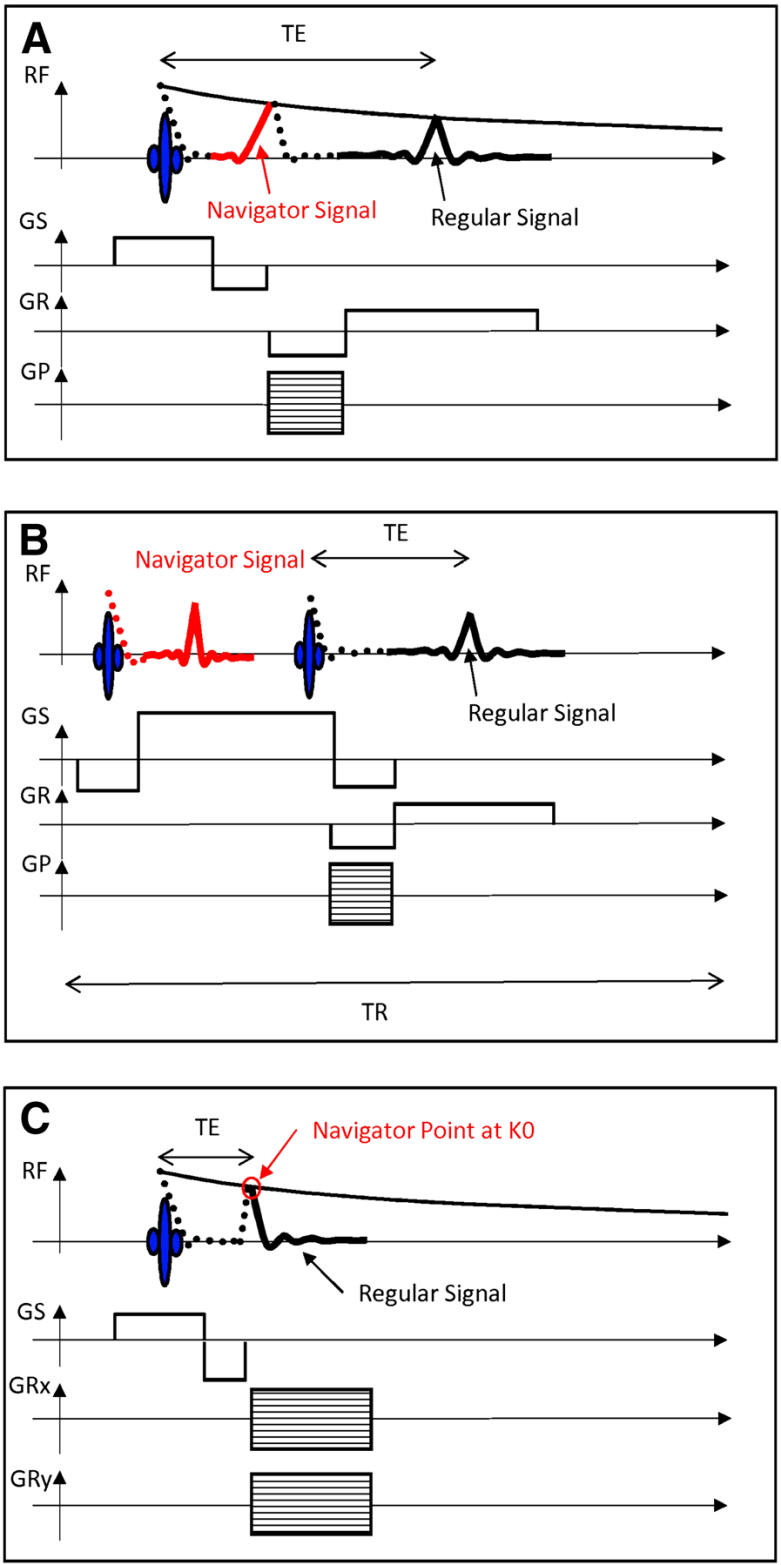
**Illustration of the recorded navigator in self-gated cine FLASH and UTE sequences.** In the self-gated cine FLASH sequence **(A,B)** the navigator is either derived from the refocusing slice signal **(A)**, which increases minimum TE, or taken from a navigator slice **(B)**, which increases minimum TR. In the self-gated UTE sequence **(C)** the first data point of each acquisition contains all motion information and is used as navigator point, without requiring additional experimental time.

For retrospective sorting of the acquired image data, self-gated CMR sequences rely on additional data that are recorded at some point during the repetition time. In the IntraGate FLASH sequence these data are recorded either during refocusing of the slice signal or by an additional navigator slice. If the navigator signal is derived from the slice signal, the phase encoding and pre-read dephase gradients need to be separated from the slice rephase gradient, which leads to a prolonged TE (Figure 1A). The excitation and recording of the additional navigator slice signal leads to longer minimum repetition time TR (Figure 1B).

In self-gated cine UTE sequences, however, the navigator signal is derived directly from the MR signal of the image acquisition. During center-out radial k-space sampling each spoke contains the central k-space in its origin, which leads to a further shortening of TE (Figure 1C).

Since the navigator signals have a constant encoding, they record all disturbances that occur during the measurement. Presuming that respiratory motion, cardiac motion and/or pulsatile blood flow occurs within the slice that is used to derive the navigator, a respiration and cardiac signal can be deduced and used for the retrospective reconstruction of a cardiac cine.

### CMR analysis

CNR as measure for image quality was calculated between the left and right myocardial wall and the ventricles for the end-diastolic and end-systolic frame as CNR = (signal intensity_blood – signal intensity_myocardium)/standard deviation_noise. Signal intensities and standard deviations were determined using the Amira software (Version 5.4.0, Visage Imaging GmbH, Berlin, Germany) as mean value over all segmented slices.

In UTE images CNR was also determined between the right myocardial wall and the adjacent tissues of lung, liver and muscle. Volumetric analysis of the left and right myocardium, and ventricles was performed using the Amira software. The regions of interest in the MR images were selected manually on the end-diastole and end-systole frame of each slice by tracing the epicardial and endocardial borders. In order to obtain the global parameters of the entire heart the volume for each frame was calculated as the sum of the area of interest in each slice multiplied by the slice thickness. SV, CO per minute and the ejection faction (EF) were calculated from the blood volume, determined in the end-systolic and end-diastolic phase.

Left ventricular (LV) and RV mass was calculated by multiplying the volume of the LV and RV myocardium by the tissue density of 1.05 g/cm^3^.

In addition to the determination of the functional cardiac parameters, the self-gated cine FLASH and UTE sequence was assessed with respect to flow artifacts (Q1), their boundary between the ventricular blood and the myocardium including the papillary muscles (Q2), susceptibility artifacts at the myocardium-lung interface (Q3), their interference with the depiction of the RV myocardium (Q4), acquisition artifacts (Q5) and the overall suitability for segmentation of the LV cavity and the myocardium using Segment (Version 1.9, Medviso AB, Lund, Sweden) and Amira software (Q6). The rating was performed in a blinded fashion using a 5-level scale from one to five (1 = best, 5 = worst). Statistical differences between both the functional and the quantitative values obtained for the two sequences were analyzed using Student *t* test. All aspects of analysis were performed by two CMR specialists independently.

## Results

### Image quality

#### Image contrast

Stacks of high resolution short-axis cardiac images covering the entire mouse heart were acquired from six healthy mice using the 2D cardiac-respiratory self-gated cine UTE sequence. Image quality was assessed with respect to CNR, and was compared with the results obtained with a conventional cartesian gradient echo (FLASH) sequence that is routinely used in CMR on mouse models. Both sequences acquired the same number of frames over the cardiac cycle with the same spatial resolution ((129 μm)^2^ in-plane; 1 mm slice) in the same acquisition time of 5 min per slice. Both sequences yielded high quality images, with CNR values above 20 (Table 1). A lower noise level was observed in the gradient echo cine images, resulting in higher CNR values throughout. However, for the UTE images excellent contrast was observed between the right myocardium and the surrounding tissue (Table 2).

**Table 1:** CNR values between the blood in the ventricles and the myocardial wall

**CNR**^**§**^	**End-diastole**^**$**^	**End-systole**^**$**^
UTE (left ventricle – left myocardium)	22.7 ± 5.3	21.1 ± 4.5
UTE (right ventricle – right myocardium)	25.5 ± 7.8	21.5 ± 5.2
FLASH (left ventricle – left myocardium)	40.8 ± 12.0	36.7 ± 11.0
FLASH (right ventricle – right myocardium)	39.6 ± 11.0	33.1 ± 9.7

^§^Contrast-to-noise ratio (CNR) was measured in the end-diastolic and end-systolic phase by self-gated FLASH and self-gated UTE sequences.

^$^Values are given as mean ± standard deviation values, averaged over six healthy C57BL/6 mice.

**Table 2:** CNR values between the right ventricular (RV) myocardium and surrounding tissues

**CNR**^**§**^	**End-diastole**^**$**^	**End-systole**^**$**^
RV myocardium - muscle	14.9 ± 3.1	14.9 ± 4.9
RV myocardium – liver	7.0 ± 2.2	6.7 ± 2.7
RV myocardium - lung	7.5 ± 3.2	7.5 ± 3.3

^§^Contrast-to-noise ratio (CNR) was measured in self-gated UTE images.

^$^Values are given as mean ± standard deviation values averaged over six healthy C57BL/6 mice.

#### Artifacts

**Figure 2:**
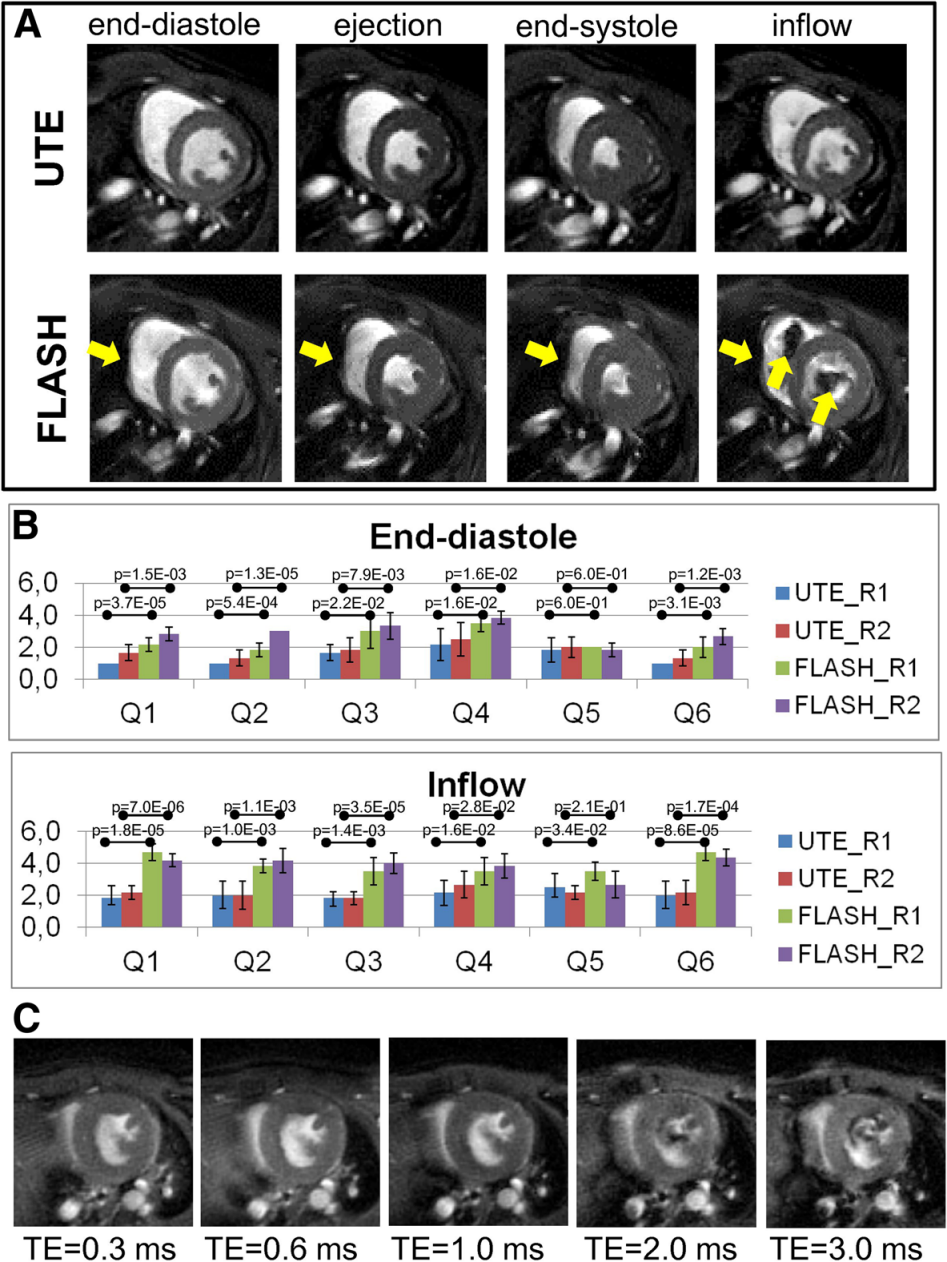
**Assessment of mid-ventricular short-axis views of a mouse heart in different cardiac phases. (A)** Exemplary self-gated cine FLASH and UTE images in the end-diastolic, ejection, end-systolic and inflow phase of a cardiac cycle. Flow and susceptibility artifacts were more pronounced in self-gated FLASH images than in UTE images and are highlighted by yellow arrows. **(B)** Results of the qualitative assessment of both self-gated UTE and FLASH images in the end-diastolic and inflow phase, performed by two independent readers (R1 and R2) using a 5-level scale (1 = best, 5 = worst). The image quality was assessed with respect to flow artifacts (Q1), their boundary between the ventricular blood and the myocardium including the papillary muscles (Q2), susceptibility artifacts at the myocardium-lung interface (Q3), their interference with the depiction of the RV myocardium (Q4), acquisition artifacts (Q5) and the overall suitability for segmentation of the LV cavity and the myocardium using Segment and Amira software (Q6). Results were averaged over images of six healthy C57BL/6 mice. (**C**) Self-gated UTE images in the cardiac inflow phase acquired with echo times of 0.314 ms, 0.6 ms, 1 ms, 2 ms, 3 ms.

Figure 2A shows four different frames of cine UTE and FLASH images during the cardiac cycle, exemplified in one central short-axis slice. To assess the image quality of the two sequences at different cardiac phases two independent readers rated images at the end-diastolic, ejection, end-systolic and the inflow phase with respect to six quality criteria. Results of the qualitative assessments in the end-diastolic and inflow phase are summarized in Figure 2B. Similar to previous studies [8,25] flow artifacts were in particular observed in the gradient echo images during the inflow phase (Q1: reader 1 [R1]: p = 0.000018, reader 2 [R2]: p = 0.000007). Further, pronounced susceptibility artifacts were observed in the T_2_*-weighted acquisitions in all cardiac phases, in particular at tissue interfaces which affected mostly the right myocardium in proximity to the lung (end-diastole: Q3: R1:p = 0.022, R2:p = 0.0079; ejection: Q3: R1:p = 0.0056, R2:p = 0.000046; end-systole: Q3: R1:p = 0.0012, R2:p = 0.0046; inflow: Q3: R1:p = 0.0014, R2:p = 0.000035). The importance of minimized TE is illustrated in Figure 2C. Longer TE, as were required in sequences with echo formation, yielded images with substantially stronger flow artifacts.

### Functional and morphological characterization

#### Cardiac function

LV and RV heart function was assessed on the basis of the cardiac images acquired with either the T_2_*-weighted FLASH or the UTE sequence. LV and RV myocardium as well as cavity were manually segmented and myocardial mass, SV, EF and CO were calculated. The LV morphological and functional parameters measured with self-gated UTE perfectly reproduced the values measured with the self-gated gradient echo method. For the RV parameters slightly but not significantly increased values were observed for myocardial mass (p = 0.10) and EF (p = 0.34) (Table 3). Bland-Altman analysis for left and right ventricular mass, SV, CO, and EF suggested high reproducibility and negligible bias of the data (not shown). LV and RV cardiac function was also investigated by 2D echocardiography as the clinical reference. Cardiac parameters obtained by echocardiography and CMR showed excellent agreement for the myocardial mass, SV and CO, but differed significantly for the EF (echocardiography vs. FLASH, p = 0.000022).

**Table 3:** Results of left and right ventricular (LV, RV) functional analysis performed by the two CMR sequences, self-gated FLASH and self-gated UTE as well as by echocardiography

	**Echocardio-graphy**^**§**^	**CMR Self-gated cine FLASH **^**§**^	**CMR Self-gated cine UTE **^**§**^	**p-values FLASH-UTE/FLASH-Ultrasound**^**§**^
**LV morphology and function**				
Mass [mg]	91 ± 11	83.7 ± 5.8	83.6 ± 3.0	0.98/0.20
Stroke volume (SV) [μl]	35.0 ± 5.4	32.3 ± 6.9	32.3 ± 6.5	0.99/0.47
Ejection fraction (EF) [%]	54.7 ± 4.8	72.2 ± 3.2	71.8 ± 2.2	0.78/0.000022
Cardiac output (CO) [ml/min]	16.6 ± 3.2	15.6 ± 3.8	15.6 ± 3.8	0.99/0.63
**RV morphology and function**				
Mass [mg]	-	15.0 ± 4	19.3 ± 3.7	0.10
Stroke volume (SV) [μl]	31.7 ±7.5 (Doppler)	22.9 ± 3.5	22.6 ± 3.9	0.89/0.026
Ejection fraction (EF) [%]	-	72.4 ± 6.1	69.6 ± 3.2	0.34
Cardiac output (CO) [ml/min]	13.2 ± 3.2 (Doppler)	11.0 ± 2.2	10.9 ± 2.1	0.89/0.20

^§^Values are given as mean ± standard deviation values averaged over six healthy C57BL/6 mice.

#### Morphology

**Figure 3:**
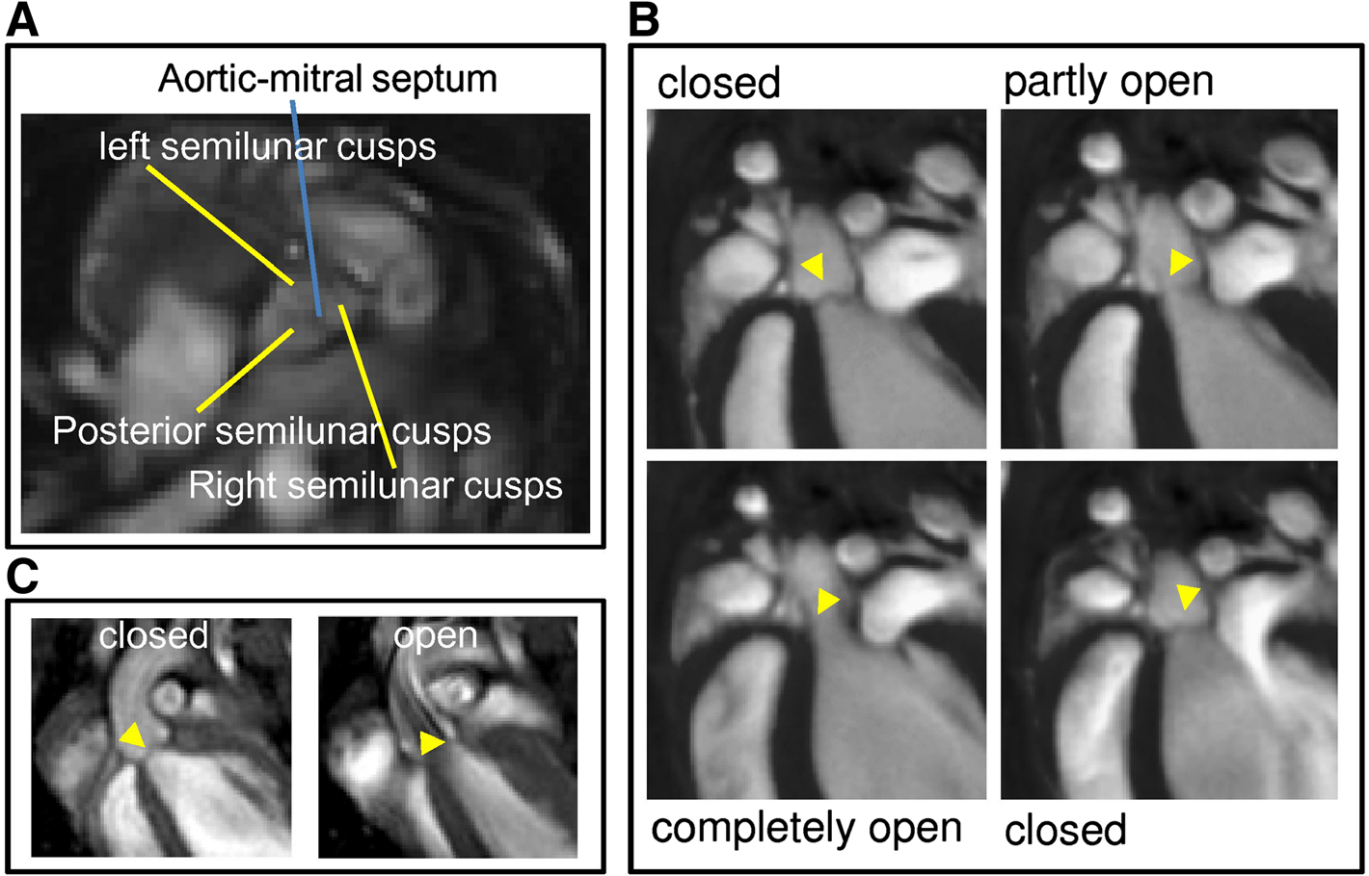
**Self-gated cine FLASH and UTE images of the aortic valve.** Self-gated UTE images **(A)** in short-axis orientation visualizing the aortic-mitral septum and **(B)** long-axis view of the aortic valve (yellow arrowhead) in different states during the heart cycle. **(C)** Self-gated FLASH images of the aortic valve (yellow arrowhead) in closed and open position.

As shown in Figure 2A images acquired with self-gated UTE were virtually free from artifacts and provided high tissue contrast, which allowed for detailed visualization of anatomical structures of the aortic valves (Figure 3A). Left, right and posterior semilunar cusps were clearly separated by the aortic-mitral septum. While flow artifacts in conventional T_2_ and T_2_* weighted images hampered the visualization of the aortic valves in open position (Figure 3C), UTE images allowed for visualization of the cardiac valves over the entire cardiac cycle (Fig ure 3B).

## Discussion

We have implemented and used a self-gated UTE sequence for assessment of functional cardiac parameters in mice at a magnetic field strength of 9.4 T. The center-out acquisition scheme allowed for a TE of only 314 μs, which minimized typical high field problems like flow and susceptibility artifacts. A further reduction in TE can be achieved when a half-pulse excitation is used [26,27]. However, since two scans have to be added pairwise, even small displacements from cardiac motion may lead to additional artifacts. Furthermore, half-pulse excitation doubles total scan time. We found that already without half-pulse excitation, a TE of 314 μs provided sufficient image quality, TE could be reduced substantially compared to self-gated cartesian sampling schemes, because no time for echo formation and acquisition of navigator data was required. Also compared to a previous implementation of self-gated radial acquisition, TE was reduced by a factor of four [18,19], and also radial FLASH acquisitions [28-30] will not be able to reach TE values as short as reported here. Due to the increased artifacts at longer TE (see Figure 2C) we did not compare our method with radial FLASH, although a reduction in scan time would have been possible.

As expected, the minimized TE almost completely suppressed flow and susceptibility artifacts (Figure 2) and thus confirmed the robustness of UTE methods in the presence of flow [11]. Although excellent image quality can be obtained by cartesian sampling schemes at even higher magnetic fields [31-38], we found that identification of small morphological structures like valves or papillary muscles was improved by using the self-gated UTE sequence. With UTE small structures were visualized in great detail, although the SNR was reduced by a factor of two, compared to self-gated FLASH. In particular for the application of automated segmentation algorithms, the improved image quality may outbalance the lower SNR [39]. The reduced SNR in the UTE images stems from two origins, which each is responsible for roughly a factor square-root of two reduction. To achieve the same scan time with UTE, polar undersampling by a factor of two was chosen, and further, FLASH data could be acquired with a slightly lower bandwidth (75 kHz vs. 100 kHz).

For assessment of cardiac functional indices it has previously been found that self-gating sequences yield comparable values to those obtained with prospective triggering [9]. Our data confirm that indices obtained with both self-gated UTE and self-gated FLASH agree perfectly with data from echocardiography (Table 3). The systematic overestimation of the EF by CMR has been observed in accordance with previous reports [40]. One great advantage of self-gated data acquisition and any other restrospective reconstruction in general is that temporal resolution (i. e. number of cardiac frames) has not to be fixed at the beginning of the measurement [41,42]. As long as sufficient repetitions have been recorded the number of reconstructed frames is only limited by the resulting SNR. As recently shown, temporal resolutions of nearly 1 ms can be achieved, which allows for assessment of diastolic dysfunction [43], a condition that requires elaborate experimental design if studied with prospectively triggered CMR [40].

A recent study has found that self-gated sequences are more robust and more efficient for imaging of the aorta in mice [25]. However, prospective methods have higher demands on the ability, experience and diligence of the experimenter. Slightly imperfect placement of the electrodes or adjustment of scan parameters may have great impact on image quality. Self-gating methods, on the opposite, have proven to be very robust. While for small animal CMR this issue is less critical, it might be for ultra-high field CMR on human subjects. There, self-gating avoids problems in ECG-signal analysis that have previously been found to be critical [44], and do not require the use of alternative gating methods. Besides the convenient and efficient use for accurate characterization of mouse models of cardiac disease, we therefore see potential for self-gated UTE in clinical application at ultra-high field.

## Conclusion

In conclusion, we have shown that self-gated UTE is a reliable and accurate technique in CMR and allows for robust and versatile measurement of most cardiac parameters of diagnostic interest. The sequence provided high quality images and compared to self-gated FLASH, susceptibility and flow artifacts were significantly decreased in particular during the inflow phase of the cardiac cycle due to the use of short TE. Enhanced image quality allowed for reliable quantification of both the left and right ventricular function and made the visualization of small anatomical structures such as the papillary muscles and the cardiac valve possible during one cardiac cycle.

## Competing interests

The authors VH, NN, MK, JS and CF declare that they have no competing interests. AN is an employee of Bruker BioSpin MRI GmbH.

## Authors’ contributions

VH coordinated the study, designed experiments, performed CMR, collected and analyzed data and wrote the manuscript, NN designed experiments, performed CMR and analyzed data, AN wrote and implemented the self-gated UTE pulse sequence program, MK designed experiments, JS performed echocardiography and analyzed data, CF designed experiments and wrote the manuscript. All authors edited the manuscript. All authors read and approved the final manuscript.
